# Split-VMAT technique to control the deep inspiration breath hold time for breast cancer radiotherapy

**DOI:** 10.1186/s13014-021-01800-x

**Published:** 2021-04-20

**Authors:** Sara Poeta, Younes Jourani, Alex De Caluwé, Robbe Van den Begin, Dirk Van Gestel, Nick Reynaert

**Affiliations:** 1 Medical Physics Department, Institut Jules Bordet – Université Libre de Bruxelles, Brussels, Belgium; 2 Radiation Oncology Department, Institut Jules Bordet - Université Libre de Bruxelles, Brussels, Belgium

**Keywords:** Split-VMAT, Deep inspiration breath-hold, SGRT

## Abstract

**Background:**

To improve split-VMAT technique by optimizing treatment delivery time for deep-inspiration breath hold (DIBH) radiotherapy in left-sided breast cancer patients, when automatic beam-interruption devices are not available.

**Methods:**

Ten consecutive patients were treated with an eight partial arcs (8paVMAT) plan, standard of care in our center. A four partial arcs (4paVMAT) plan was also created and actual LINAC outputs were measured, to evaluate whether there was a dosimetric difference between both techniques and potential impact on the delivered dose. Subsequently, ten other patients were consecutively treated with a 4paVMAT plan to compare the actual treatment delivery time between both techniques. The prescribed dose was 40.05 Gy/15 fractions on the PTV breast (breast or thoracic wall), lymph nodes (LN) and intramammary lymph node chain (IMN). Treatment delivery time, PTVs coverage, conformity index (CI), organs at risk (OAR) dose, monitor units (MU), and gamma index were compared.

**Results:**

Both split-VMAT techniques resulted in similar dose coverage for the PTV Breast and LN, and similar CI. For PTV IMN we observed a 5% increased coverage for the volume receiving ≥ 36 Gy with 4paVMAT, with an identical volume receiving ≥ 32 Gy. There was no difference for the OAR sparing, with the exception of the contralateral organs: there was a 0.6 Gy decrease for contralateral breast mean (*p* ≤ 0.01) and 1% decrease for the volume of right lung receiving ≥ 5 Gy (*p* = 0.024). Overall, these results indicate a modest clinical benefit of using 4paVMAT in comparison to 8paVMAT. An increase in the number of MU per arc was observed for the 4paVMAT technique, as expected, while the total number of MU remained comparable for both techniques. All the plans were measured with the Delta^4^ phantom and passed the gamma index criteria with no significant differences. Finally, the main difference was seen for the treatment delivery time: there was a significant decrease from 8.9 to 5.4 min for the 4paVMAT plans (*p* < .05).

**Conclusions:**

This study is mainly of interest for centers who are implementing the DIBH technique without automatic beam-holding devices and who therefore may require to manually switch the beam on and off during breast DIBH treatment. Split-VMAT technique with 4 partial arcs significantly reduces the treatment delivery time compared to 8 partial arcs, without compromising the target coverage and the OAR sparing. The technique decreases the number of breath holds per fraction, resulting in a shorter treatment session.

## Introduction

In the last 15 years, considerable efforts have been made to minimize cardiac and lung toxicity of postoperative radiotherapy for left-sided breast cancer. The implementation of techniques such as intensity modulated radiotherapy and deep inspiration breath hold (DIBH) allowed for a better sparing of these organs at risk (OARs) [1–6]. Literature suggests that the combination of volumetric modulated arc therapy (VMAT) and DIBH can even further decrease the mean heart dose and the ipsilateral lung dose for left-sided breast cancer radiotherapy including regional lymph nodes and IMN [7–9].

Different treatment planning solutions to combine DIBH and VMAT are described in the literature, including the use of multiple small partial arcs (split-VMAT) that mimic tangential fields; a full 360° arc; a hybrid plan combining tangential fields and partial arcs; and a single or double partial arc totaling between 190° and 250° [6–14]. In the last few years, multiple dosimetric studies have been published comparing these different VMAT treatment designs for left-sided DIBH, showing better results for split-VMAT techniques, regardless whether nodal areas had to be treated as well [13, 15–18].

The increasing use of surface guided radiation therapy (SGRT) systems has allowed for a reduction localization uncertainty during treatment delivery. When using DIBH techniques, SGRT enables monitoring of the patient movements and respiration, optimizing position reproducibility and minimizing internal target motion, hence increasing the accuracy of the treatment of specific anatomic sites [19–21]. This system can be linked to the treatment machine to trigger automatic beam delivery or beam hold when respiration is within predefined respiratory phases [20]. When SGRT or any other monitoring device is not present or linked to the beam hold, RTTs have to manually interrupt the beam by observing the patient’s respiration. This can be uncomfortable for the RTTs and might create insecurity with the technique, mainly during the implementation of the technique. Hence, we decided to apply split-VMAT arcs with planned stops to systematize the treatment for both patients and RTTs, allowing them to minimize unplanned beam interruptions during treatment.

We designed a technique using 8 partial arcs (8paVMAT) with a 20 s maximum treatment time per arc. This solution led to a long treatment time per fraction given 8 separate DIBH were necessary to be able to deliver all the arcs. Based on feedback from patients and RTTs revealing that a majority of patients were able to comfortably hold their breath more than 20 s, we decided to reduce the number of arcs to four arcs with an average delivery time of 30 s per arc (4paVMAT). In case the breath hold had to be interrupted during the treatment, the RTTs could stop the beam manually and start the treatment again where it was interrupted.

In this work, we compare the 4paVMAT technique with the 8paVMAT and its benefits in terms of treatment time.

## Methods and materials

### Treatment planning techniques

**Fig. 1:**
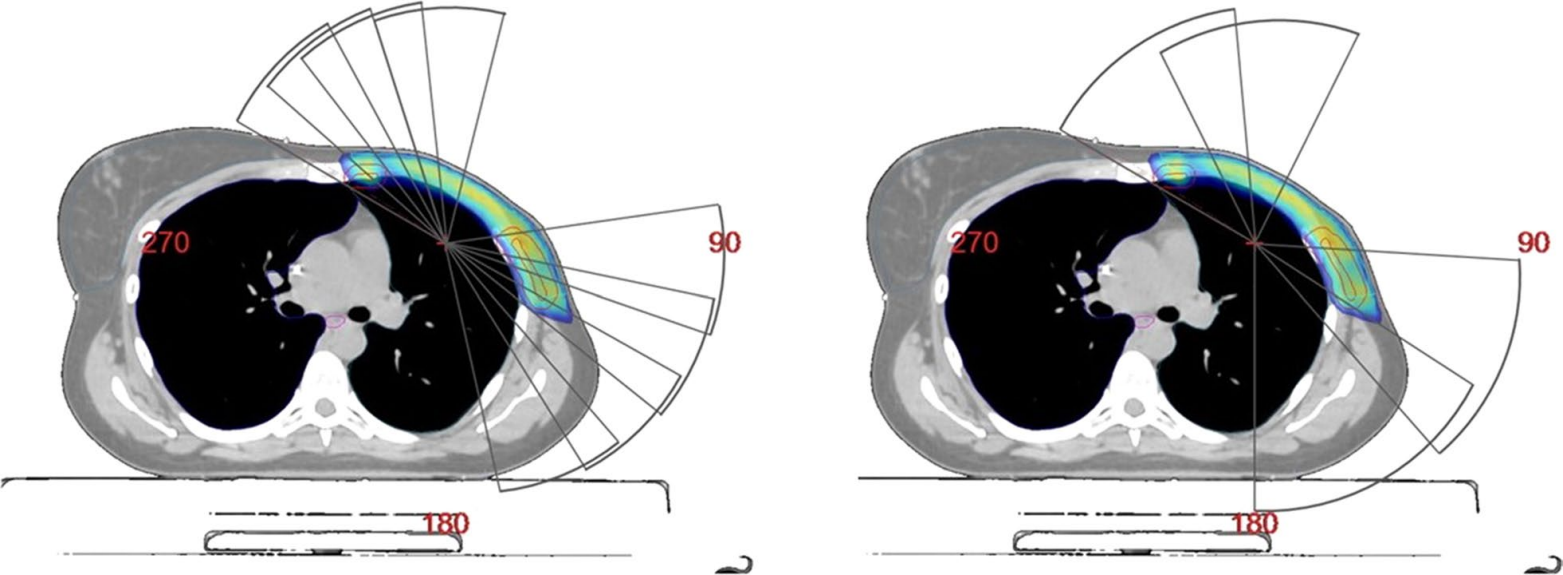
Axial caption of the treatment arcs set up for one of the selected patients. On the left the 8paVMAT is displayed and on the right the 4paVMAT

Two different planning techniques are compared in this study: a 8 partial arcs VMAT (8paVMAT) and a 4 partial arcs VMAT (4paVMAT).In an 8paVMAT plan, 30° overlapping arcs mimic tangential fields, where the start/stop angle was between 300°/20° for the medial arcs and between 80°/180° for the lateral ones. In 4paVMAT, the angle of the arcs was increased to 50° keeping the start/stop angle between 300°/20° for the medial arcs and between 90°/180° for the lateral arcs, allowing the arcs to overlap in both techniques (see Fig. 1).

All plans were generated with Monaco v5.11 treatment planning system (Elekta AB, Stockholm, Sweden), with 6MV photons. The number of control points (CP) per arc was also increased in the 4paVMAT setting. The gantry angle increment was decreased, to increase the modulation. This value correlates with the number of sectors during the first optimization step in which the multileaf collimator moves continuously from one side to the other and then changes its direction.

The collimator angles were 15° and 345° for the different arcs, in order to be parallel to the chest and to the heart. A virtual bolus of 5 mm was used for the planning and removed at the end of the optimization to take into account the breast movement and possible swelling between fractions [22–24]. For all patients, 40.05 Gy in 15 fractions was prescribed to the breast or thoracic wall, LN and IMN. All plans were normalized at 95% of the breast PTV to receive 95% of the prescribed dose (38.05 Gy).

Table 1 shows the different parameters used for both templates during the optimization.

**Table 1:** Parameters defined for 8paVMAT and 4paVMAT plans

Monaco TPS parameters	8paVMAT	4paVMAT
*Objective*		
Delivery time per arc (s)	20	30
*Parameters*		
number of arcs	8	4
number of CPs per arc	13–16	80–120
Arc length	30°	50°
Limits of medial arcs	300°–20°	300°–20°
Limits of lateral arcs	80°–180°	90°–180°
Angle increment	30°	20°

*CP* control points

### Dosimetric and QA comparisons

- Patient selection

Left sided breast cancer patients necessitating intramammary lymph node (IMN) irradiation or patients with a challenging anatomy for which VMAT was deemed necessary were included. All patients were treated using DIBH with real-time monitoring using SGRT.

Ten breast cancer patients, consecutively treated in our center with VMAT and DIBH using an 8paVMAT plan, were selected for the present study. For every patient, an additional 4paVMAT plan was subsequently created in order to investigate target coverage and dose to the OAR.

(b) Plan comparison

The twenty plans were evaluated and compared. PTV’s coverage, OARs dose and number of monitor units (MU) were compared between plans. The D_90%_ coverage for all PTVs and the conformity index (CI) were compared. The CI is defined as CI = TV_*RI*_/TV * TV_*RI*_/V_*RI*_, where TV_*RI*_ is the target volume covered by the reference isodose (95% of the prescribed dose), TV is the target volume and V_*RI*_ is the volume encompassed by the reference isodose [25]. For OARs, mean heart doses, V_17*Gy*_ and V_5Gy_ to the heart, mean contralateral breast doses, V_17*Gy*_ for ipsilateral lung, mean lungs doses, V_5Gy_ to the contralateral lung and body were compared. The choice of these constraints was based on an internal protocol which regroups constraints found in the literature [26–30]. The total MU, and the minimum and maximum MU per arc were also compared. “DVH Analytics” was used for plan comparison [31].

(iii) Plan measurements

Actual LINAC output was measured to evaluate whether there was a dosimetric difference between both techniques and if it would have an impact on the delivered dose.

For Quality Assurance (QA), the gamma index was used, which provides a numerical quality value that serves as a measure of disagreement in the regions that fail the acceptance criteria [32]. All the plans were measured on an Elekta Infinity^*TM*^ equipped with an Agility^*TM*^ head with the Delta^4^ + phantom using global gamma evaluation with 3 %/3 mm criteria above a 20% of maximum dose threshold for 95% of measured points.

In cases where patients cannot hold their breath long enough, the RTTs interrupt the beam manually and restart the treatment where it was stopped. To ensure correct treatment delivery in case of beam interruption, two QA measurements were acquired for 4paVMAT: one without interruptions and another interrupting every arc.

#### Treatment delivery time comparison

The ten patients of the dosimetric study were treated with the 8paVMAT plan and treatment times were recorded. After validation of the 4paVMAT technique by comparing DVH parameters and QA in these patients, ten new consecutive patients were included, planned and treated with the 4paVMAT technique. Treatment times were compared with the 8paVMAT treatment times.

### Contouring and treatment details

The clinical target volumes (CTVs) were delineated according to ESTRO guidelines and a margin of 5mm was added around the CTV to obtain the planning target volume (PTV) for breast or thoracic wall, lymph nodes (LNs) and IMN. The PTVs were cropped at 3mm from the surface of the skin. SGRT with the IDENTIFY system from Humediq (Varian Medical Systems, Palo Alto, CA, USA) was used to optimize set up and monitor DIBH.

### Statistical analysis

The statistical analysis was performed using a Mann-Whitney U test for treatment delivery time, a Wilcoxon signed-rank test for all the other parameters, and Friedmann test for the gamma index criteria evaluation, at a significance level under 0.05.

## Results

Patient characteristics are shown in Table 2. Patients underwent breast conserving surgery or mastectomy and the mean age for 8paVMAT and 4paVMAT treated groups is 45.7 and 60.6 years old, respectively.

**Table 2:** Treated volumes for the patients selected

Treated volume	Number of patients	
	8paVMAT (n = 10)	4paVMAT (n = 10)
Breast/thoracic wall	2/8	3/7
LN I	4	5
LN II	8	7
LN III	10	10
LN IV	10	10
Rotter	6	6
IMN	10	10

LN: lymph node level, IMN: internal mammary nodes

Table 3 shows the dosimetric results from the two different techniques.

**Table 3:** Dosimetric parameters results obtained for 8paVMAT and 4paVMAT (data are shown as mean values with one standard deviation, and range between brackets)

Structure	Objective/constraint	8paVMAT	4paVMAT	*p* value
PTV breast D_90%_ (Gy)	Constraint ≥ 38.05	38.9 ± 0.17 [38.7–39.2]	38.9 ± 0.18 [38.7–39.3]	0.17
PTV breast CI	–	0.61 ± 0.10 [0.45–0.76]	0.60 ± 0.10 [0.43–0.71]	0.10
PTV IMN V_32Gy_ (%)	Constraint ≥ 90	95.6 ± 2.46 [91.4–100]	96.4 ± 1.99 [93.8–100]	0.41
PTV IMN V_36Gy_ (%)	Objective ≥ 90	78.8 ± 10.4 [60–96.6]	84.2 ± 5.7 [77.5–97.4]	*0.037*
PTV LN V_36Gy_ (%)	Constraint ≥ 90	95.9 ± 3.03 [91.8–99.4]	97.5 ± 1.79 [93.6–99.6]	0.20
Heart D_Mean_ (Gy)	Objective ≤ 4	2.8 ± 0.9 [1.55–4.6]	2.9 ± 0.9 [1.8–4.6]	0.92
Heart V_5Gy_ (%)	Objective ≤ 10	9.6 ± 3.4 [3.3–17.1]	9.8 ± 3.3 [3.2–16.6]	0.72
Heart V_17Gy_ (%)	Constraint ≤ 10	2.4 ± 2.5 [0.0–7.6]	2.6 ± 2.5 [0.0–8.0]	0.06
Ipsilateral Lung V_17Gy_ (%)	Constraint ≤ 35	25.2 ± 3.4 [18.6–29.2]	25.3 ± 3.8 [17.7–30]	0.68
Lungs D_Mean_ (Gy)	Objective ≤ 6	5.9 ± 0.6 [4.7–6.5]	5.8 ± 0.4 [5.1–6.4]	0.16
C. Lung V_5Gy_ (%)	–	3.6 ± 2.1 [1.5–7.7]	2.4 ± 2.0 [0.3–6.2]	*0.024*
C. Breast D_Mean_ (Gy)	Objective ≤ 3.5	3.6 ± 0.7 [1.9–4.7]	3.0 ± 0.7 [1.5–4.0]	≤ * 0.01*
Humeral head PRV V_30Gy_ (%)	Objective ≤ 2	6.3 ± 6.9 [0.0–21.2]	8.1 ± 9.1 [0.0–26.3]	0.15
Body V_5Gy_ (cc)	–	5376 ± 1241 [3359.6–7400]	5396.9 ± 1301.2 [3153.5–7485]	0.96

Italic emphasis reflects the significant results

PRV—planning organ at risk volume, CI—conformity index, C. Breast—contralateral breast, C. Lung—contralateral lung

**Fig. 2:**
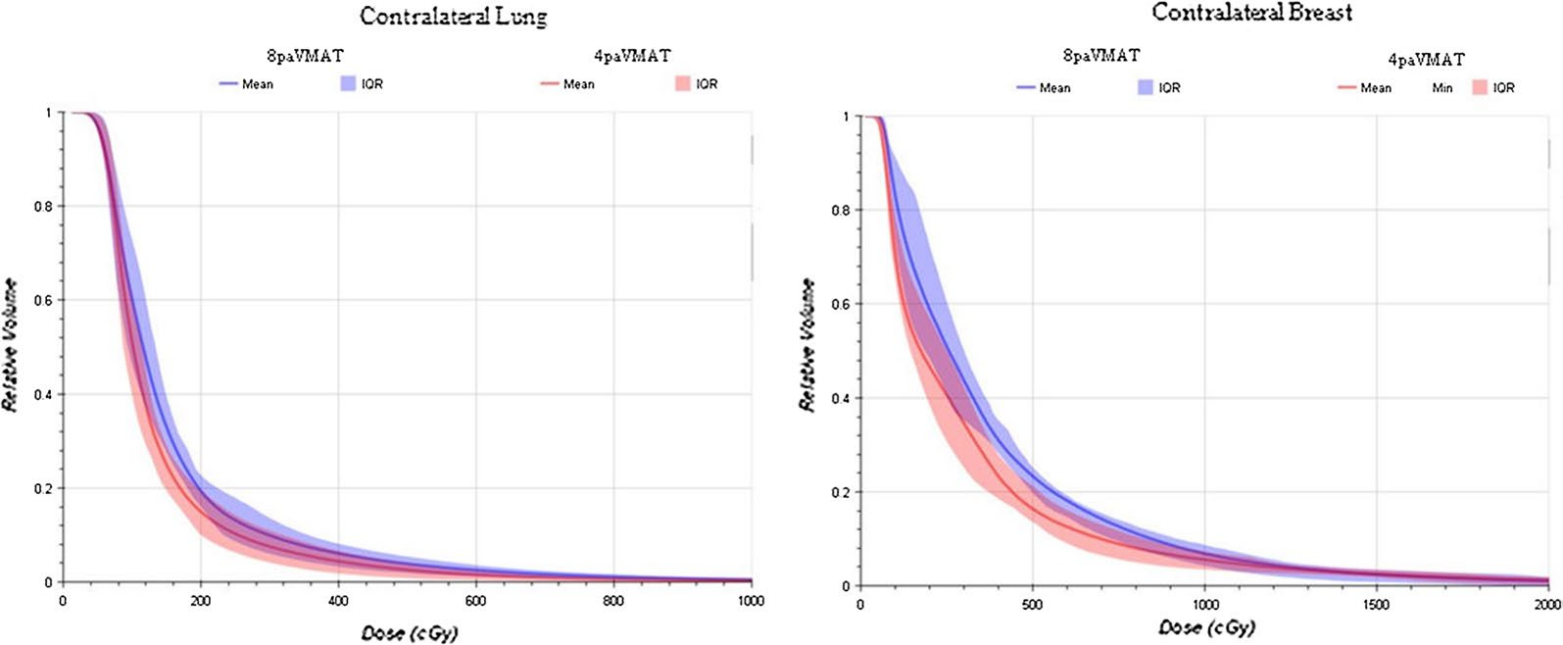
The mean DVH for right lung (left figure) and contralateral breast (right figure) using 8paVMAT and 4paVMAT. The colored shadows show the interquartile ranges (IQRs) from the mean values

**Fig. 3:**
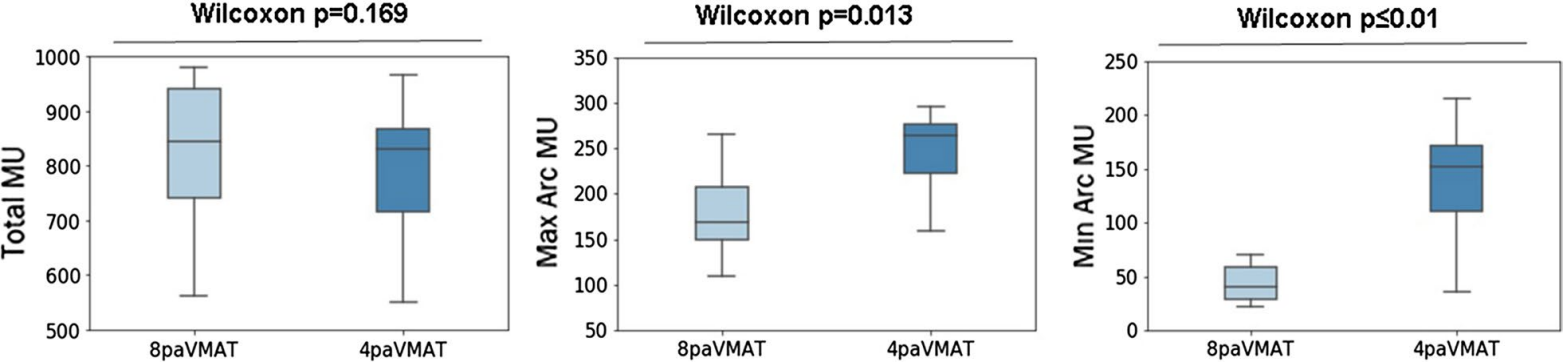
Boxplots of MU for 8paVMAT and 4paVMAT (total MU of all arcs, maximum MU per arc, minimum MU per arc)

Both techniques met the mandatory dose constraints for OAR and target coverage. We can observe similar results for the PTV breast and PTV LN coverage, and conformity index. A significant difference is observed for the PTV IMN 36 Gy coverage: there is a 5.4% coverage increase with 4paVMAT plans. For the OARs, both techniques showed similar results for the main constraints: there was no difference in mean heart dose, V_5*Gy*_ and V_17*Gy*_ to the heart, mean lung doses, V_17Gy_ to the ipsilateral lung, V_30*Gy*_ to the humeral head and V_5*Gy*_ to the body. Regarding the doses to the contralateral organs, there is an average decrease of 0.6 Gy (*p* < 0.01) for the mean contralateral breast dose and 1.2% (*p* = 0.024) for the volume of contralateral lung receiving > 5 Gy, with the 4paVMAT plans. Figure 2 shows the mean dose-volume histograms (DVH) for contralateral lung and contralateral breast. There is no difference in the total number of MU between both treatments, but we see a significant, yet logical increase of the minimum and maximum MU per arc with 4paVMAT—see Fig. 3 (*p* < 0.05).

**Fig. 4:**
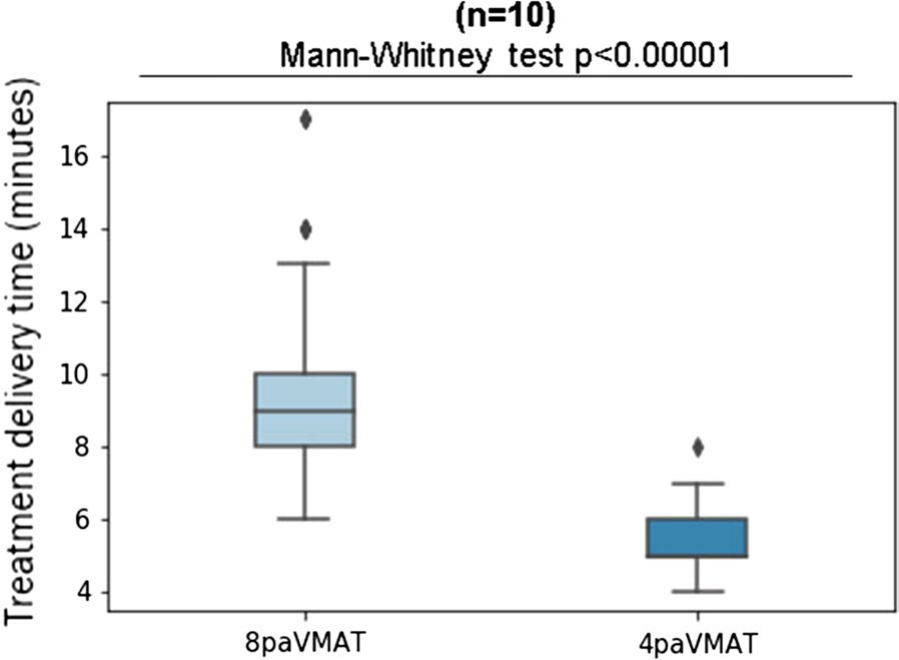
Mean treatment delivery time in minutes for 8paVMAT and 4paVMAT

**Fig. 5:**
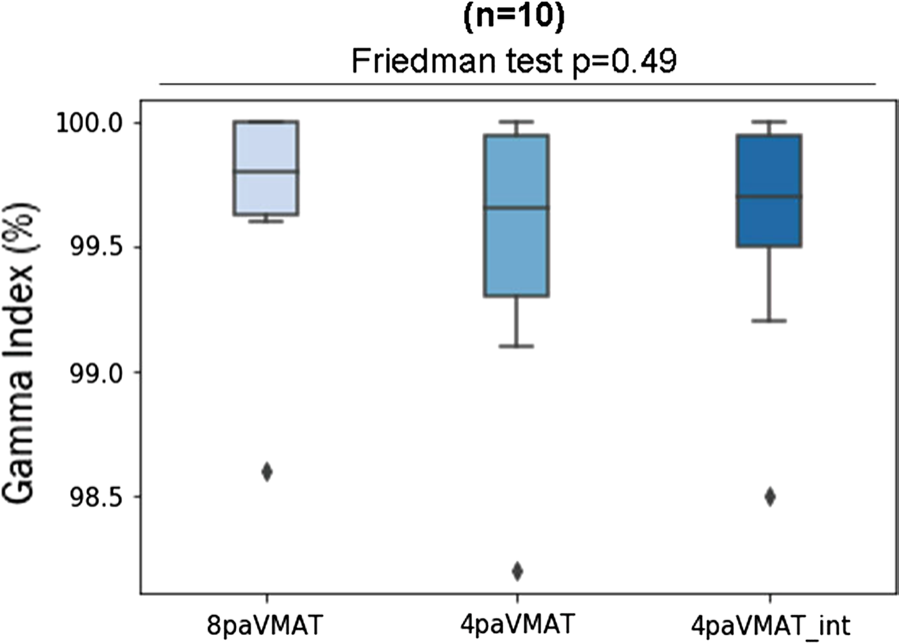
Measurement results with the gamma index for 8paVMAT and 4paVMAT with and without treatment interruption

The main difference was seen in treatment delivery time with 8.9 min and 5.4 min for 8paVMAT and 4paVMAT, respectively (*p* < 0.01)—Fig. 4. Regarding the QA measurements, all the plans passed the gamma index criteria, whether the arcs were interrupted or not. The results can be seen in Fig. 5.

## Discussion

Previous dosimetric studies have shown exciting achievements regarding the constraint goals for OAR with split-VMAT techniques [13, 15–17, 33]. However, very few studies focused on the split-VMAT technique itself [13].

In our institute, the combination of VMAT and DIBH for left-sided breast treatment was introduced using split-VMAT with 8 partial arcs. This design was chosen to systematize the patient’s treatment and to decrease the number of unplanned beam interruptions. We aimed to decrease the treatment time per fraction and decided to compare DIBH 8paVMAT with 4paVMAT.

The combination of the optimization parameters of the 4paVMAT resulted in an adequate plan with a decreased treatment delivery time per fraction. Our analysis reveals that 8paVMAT and 4paVMAT demonstrate equivalent coverage of the PTV Breast and LN, and the CI. Although there is no difference for the PTV IMN regarding the volume receiving 32 Gy, there is a significant 5% increase of the volume receiving 36 Gy, when using 4paVMAT.

Our results are consistent with other dosimetric studies regarding target coverage and OARs sparing [7, 13, 34, 35]. Table 4 compares published data with our results.

**Table 4:** Comparison of average dose parameters for VMAT with DIBH between different dosimetric studies

	8paVMAT	4paVMAT	Osman [7]	Ranger [34]	Rossi [35]
Prescription	40 Gy/15x	40 Gy/15x	42.56 Gy/16x	40 Gy/15x	50 Gy/25x
*Structure*					
Heart D_*Mean*_ (Gy)	2.8 ± 0.9	2.9 ± 0.9	4.1 ± 1.4	2.6 ± 1.0	3.3 ± 0.9
Heart V_5*Gy*_	9.6 ± 3.4	9.8 ± 3.3	–	–	12.8 ± 5.1
Heart V_17*Gy*_	2.4 ± 2.5	2.6 ± 2.5	–	1.8 ± 2.1	–
Ipsilateral lung V_17*Gy*_ (%)	25.2 ± 3.4	25.3 ± 3.8	–	28.2 ± 4.5	–
Lungs D_*Mean*_ (Gy)	5.9 ± 0.6	5.8 ± 0.4	7.5 ± 1.4	–	–
C. Lung V_5*Gy*_ (%)	3.6 ± 2.1	2.4 ± 2.0	11.2 ± 6.7	–	8.4 ± 9.0
C. Breast D_*Mean*_ (Gy)	3.6 ± 0.7	3.0 ± 0.7	2.5 ± 1.0	1.5 ± 0.9	3.9 ± 2.2

C. Breast—contralateral breast, C. Lung—contralateral lung

The main differences may be due to differences in prescription, structure margins or OAR priority.

For the OARs, there was no significant difference between 4 and 8paVMAT with respect to the mean heart doses, heart V_5Gy_, heart V_17Gy_, ipsilateral lung V_17*Gy*_, mean lung doses, V_30*Gy*_ of the humeral head PRV and V_5Gy_ to the body.

In our study, there was an improvement of 0.6 Gy in the mean contralateral breast doses (*p* < 0.01) for the 4paVMAT plan. There was also a significant 1% decrease for the volume of contralateral lung receiving 5 Gy. One possible explanation for this is the increased scattered dose with 8paVMAT because of the number of arcs [36]. Yet, further studies should be performed to confirm these results. These are the small dosimetric benefits of the 4paVMAT template.

Many concerns have been raised regarding the low dose bath to peripheral organs and the increased risk for radiation induced malignancy or the still unknown effects of such doses. Our results show the volume of contralateral lung receiving 5 Gy of 3.6% and 2.4% for 8paVMAT and 4paVMAT, respectively. These results show that the low dose volumes can be comparable between VMAT and 3D CRT techniques, the latter being well known for a very low to almost no dose to the contralateral lung.

The observed increase in treatment time per arc resulted, as expected, from an increase of the minimum and maximum number of MU per arc with 4paVMAT, however no difference in the total number of MUs between both techniques was found.

During our QA checks, beam interruption was simulated for 4paVMAT technique for all arcs to ensure that the treatment maintained a good deliverability (4paVMAT *int*). Figure 5 shows that all the checks, even those with a beam interruption, passed the global gamma index evaluation, using 3%/3 mm criteria above a 20% of maximum dose threshold for more than 95% of the measured points. This proves the linear accelerator to reliably deliver the dose, even in case of beam interruptions.

The main goal of this study was to decrease the treatment delivery time per fraction. To the best of our knowledge, no study has been published to compare the treatment delivery time for different VMAT techniques combined with DIBH for left-sided breast radiotherapy.

The mean treatment delivery time was significantly decreased by around 40% with 4paVMAT, enabling us to spare on average 3.5 min beam-on time per fraction (Fig. 4). This also reduced the number of unplanned beam interruptions for both RTTs and patients, and proved to be in total a faster and more convenient delivery solution. In a time slot of 20 min for DIBH and 15 min for normal treatments, this means a gain of almost 20% which makes it possible to treat up to 5 more patients on an 8 h treatment day.

A limitation of the present study is the fact that the delivery time was measured in different patients for 4paVMAT and 8paVMAT plans. However, since the differences in treated volumes were small (Table 2), we expect that this would have little impact on the average treatment time.

Finally, this new delivery technique was successfully adopted in the department. The 4paVMAT did not lead to any issue regarding the breath holds by any of the patients. Whenever the patients were not able to hold their breath long enough, the RTTs interrupted the beam manually and started the treatment again where it had been stopped.

## Conclusion

This study is mainly of interest for centers who are implementing the DIBH technique without automatic beam-interruption devices, and who therefore require to manually switch the beam on and off during breast DIBH treatment. We provide a solution using a pre-planned number of beam interruptions during the treatment.

The 4paVMAT technique provides a faster radiotherapy delivery option than 8paVMAT for DIBH treatment of breast cancer including regional LN and IMN, without clinically important dosimetric differences for targets coverage and OAR sparing. With potentially half the number of breath holds per fraction, this technique enabled us to decrease the treatment time by about 3.5 minutes per fraction. QA measurements showed 4paVMAT to be correctly delivered, even in case of beam interruption; hence, it was adopted in our department as the new standard for VMAT treatment of left-sided breast cancer with DIBH.

## Data Availability

Research data are stored in an institutional repository and could be shared upon request to the corresponding author.

## Abbreviations

8paVMAT: A Eight partial arcs
4paVMAT: A four partial arcs
LN: Lymph nodes
IMN: Intramammary lymph node chain
CI: Conformity index
MU: Monitor units
OAR: Organs at risk
DIBH: Deep inspiration breath hold
VMAT: Volumetric modulated arc therapy
CP: Number of control points
SGRT: Surface guided radiation therapy
QA: Quality Assurance
CTVs: Clinical target volumes
PTV: Planning target volume
TV_*RI*_: Target volume covered by the reference isodose (95% of the prescribed dose)
TV: Target volume
V_*RI*_: Volume encompassed by the reference isodose
DVH: Dose-volume histograms
IQRs: Interquartile ranges
